# Glucocorticoid Insensitivity: Is It a Question of Time and Place?

**DOI:** 10.3390/biomedicines13061418

**Published:** 2025-06-10

**Authors:** Christopher Lambers, Michael Roth

**Affiliations:** 1 Department Pneumology, Ordensklinikum Linz Elisabethinen, AT-4020 Linz, Austria; 2 Pulmonary Cell Research, Department of Biomedicine & Clinic of Pneumology, University and University Hospital Basel, CH-4031 Basel, Switzerland; rothchiarello@gmail.com

**Keywords:** chronic inflammatory lung diseases, glucocorticoid receptor kinetics, disease specificity, cell type specificity, omics studies, artificial intelligence analysis

## Abstract

**Background:** Glucocorticoid insensitivity is a problem for the therapy of chronic inflammatory lung diseases, such as asthma and chronic obstructive pulmonary disease (COPD). Both are non-communicable chronic inflammatory lung diseases with worldwide increasing incidences. Only symptoms can be controlled by inhaled or systemic glucocorticoids, often combined with β2 agonists and/or muscarinic receptor antagonists. The therapeutic effect of glucocorticoids varies between individuals, and a significant number of patients do not respond well. It is believed that only protein-free circulating unbound glucocorticoids can enter cells by diffusion and achieve their therapeutic effect by binding to the intracellular glucocorticoid receptor (GR), encoded by the NR3C1 gene, for which over 3000 single-nucleotide polymorphisms have been described. In addition, various GR protein isoforms result from 11 transcription start sites, and differential mRNA splicing leads to further GR protein variants; each can be modified post-translational and alter steroid response. To add more variety, some GR isoforms are expressed cell-type specific or in a sub-cellular location. The GR only functions when it forms a complex with other intracellular proteins that regulate ligand binding, cytosol-to-nuclear transport, and nuclear and cytosolic action. Importantly, the timing of the GR activity can be cell type, time, and condition specific. These factors are rarely considered when assessing disease-specific loss or reduced GR response. **Conclusions**: Future studies should analyze the timing of the availability, activity, and interaction of all components of the glucocorticoid signaling cascade(s) and compare these factors between non-diseased and diseased probands, applying the combination of all omics methods (250).

## 1. Introduction

According to the 2024 GINA report, 300 million people currently suffer from asthma worldwide, causing approximately 1000 deaths per day [1]. The yearly incidence of asthma is increasing worldwide but varies when comparing different countries for many reasons, such as the lack of clear diagnostic tests, the wide variation of symptoms, overlapping symptoms with other lung diseases, and the lack of national registers in most countries [1,2]. As an example, the American Lung Association reported that between 1999 and 2023, the incidence of asthma increased by 19.1% in the USA [2]. Not only are the patients affected but the disease results in a major loss of school attendance and working hours, and it contributes to increasing health care costs and reduced quality of life for the patients. Importantly, in the wake of the COVID-19 pandemic, the incidence of chronic inflammatory lung diseases seems to increase worldwide [3].

The prevalence of chronic obstructive pulmonary disease (COPD) was estimated to have affected 392 million patients in 2019 [4]. The major risk factors for COPD were listed as male sex, smoking, and a low body mass index, as well as exposure to biomass, dust, or smoke in the environment or workplace. COPD cannot be cured; only its progress can be slowed [4,5,6].

For both diseases, asthma and COPD, glucocorticoids are often prescribed for life, with the aim of controlling inflammation. However, the response to glucocorticoids varies among patients, and this individual response, including insensitivity, cannot be predicted [5,6]. Both diseases are assumed to involve poly-genetic predispositions, which are not well understood [7]. A frequent reason for asthma might be seen in the wide variation of the genetic and post-translational modifications of the glucocorticoid receptor (GR), as we will show later. Glucocorticoids have been used for 80 years to treat inflammatory diseases, and their mode of action has been studied in detail. However, there is no method to determine or predict an individual’s steroid response prior to treatment [8]. The individual differences regarding the sensitivity to steroids are important to the beneficial as well as to the unwanted side effects of the drugs [9]. Steroid sensitivity can be modified by daytime, seasons, sex, and/or age [10,11]. Reduced or missing response to glucocorticoids was reported in nearly 30% of patients diagnosed with autoimmunity, inflammation, or malignancies [11]. In clinics, individual steroid sensitivity can present as increased side effects, reduced anti-inflammatory efficacy that limits disease control, or unaffected disease progression [12].

Moreover, the timing of GR availability can be modified by steroids and environmental conditions and thus cause temporary or lasting reduced steroid sensitivity [13,14]. Such effects have been reported for several steroids, alone or in combination with other medications, thereby modifying the patient’s response to therapy [15]. Furthermore, the transcriptional activity of the GR in response to steroids can be cell-type specific and time dependent in isolated primary cells [16,17]. However, investigations to determine, e.g., the disease-specific timing of these mechanisms, are missing.

## 2. The Glucocorticoid Receptor

**Figure 1:**
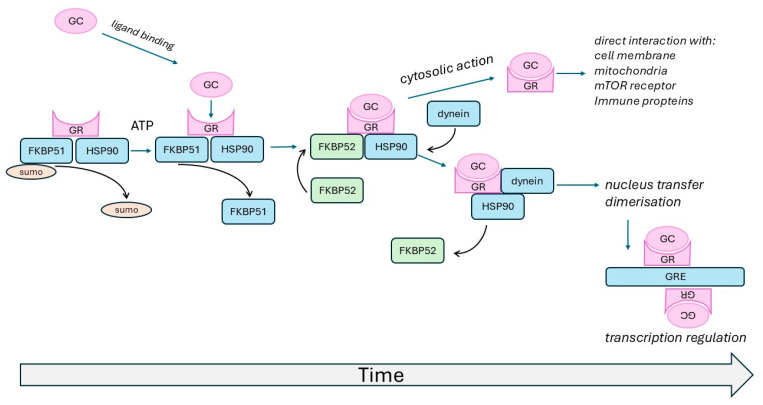
Complex formation of the GR that regulates its ligand binding, cytosolic actions, and nuclear transport with subsequent transcriptional activity. Each of these steps can be delayed and lead to reduced or missing GR function and thus steroid insensitivity. The same applies to GR ligating proteins, as has been shown for the FKBP51 gene [23,24].

The major therapeutic action of glucocorticoids is their anti-inflammatory effect, which was thought to mainly occur through their function as an intracellular transcription factor [18,19]. However, this mode of action of the GR is too simplistic. The unligated inactive GR is present in the cytosol, forming a complex with heat shock proteins (HSPs), including HSP40, HSP70, and HSP90, together with HDAC5 [20,21,22]. Upon phosphorylation, HSP40 and HSP70 dissociate from the complex and make space for FK506-binding protein 5 (FKBP51), which enables glucocorticoid binding [20]. Upon the binding of a glucocorticoid, FKBP51 is exchanged for FKBP52, which allows HSP90 to bind with dynein, leading to active transport of the GR into the nucleus (Figure 1). The strength of the interaction between the GR and FKBP51 depends on the corresponding gene alleles and feedback regulation through steroids [23,24]. The latter represents a negative feedback loop to control steroid sensitivity, which might be enhanced in patients with steroid insensitivity [24].

In the nucleus, the GR either acts as a monomer or forms a dimer with a second ligated GR. Both GR forms recognize and bind to a specific DNA sequence, named the glucocorticoid response element (GRE), which is part of many promoters of pro-inflammatory protein-encoding genes, and it down-regulates their expression [20] (Figure 1). The nuclear action of the GR has been summarized by many others and, therefore, this review will not describe the details [8,12,20,23].

The GR is encoded by the NR3C1 gene, which consists of nine exons [25]. Exon 1 contains 11 untranslated promoter variants, which can affect the transcription of the mRNAs [20]. Exons 2–9 encode the GR mRNA, which can result in at least five different mRNAs with corresponding different GR proteins [20]. Furthermore, the translation of some GR mRNAs has been reported to vary due to different translation start sites, which might be related to cell type or disease-specific translation of the GR mRNA. Overall, more than 3000 single-nucleotide polymorphisms (SNPs) of the NR3C1 gene have been reported, but the knowledge of their function or biological activity is limited [26]. Most of the NR3C1 SNPs were located in exons, which might affect the structure of the encoding DNA region and, therefore, the binding affinity of the transcription machinery, as has been described for other hormone-encoding genes [27]. Thus, some NR3C1 gene SNPs might alter the protein sensitivity to steroids in a specific individual, but even larger clinical studies on the most common NR3C1 gene SNPs are missing. Some studies discussed the possibility that NR3C1 gene SNPs have an exclusive tissue or cell-type specific effect, but these observations were mainly made in animal models, and it is an ongoing matter of debate how far animal models of the GR represent human physiology or pathophysiology [28,29,30].

## 3. The Regulation of GR Function by Time

It is well known that the effects of steroids and the function of the GR underlie a circadian control mechanism, which depends on the presence of co-regulating proteins, such as FKBP51 [31]. In addition, FKBP51 overexpression affects the circadian effect of the GR [32]. The function of the GR is also modified by circadian regulators, such as melatonin [33]. Other than in lung cells, GR activity was modulated by a circadian-regulated basement membrane structure, which might be an important factor for steroid transportation across the cell membrane and which has to be investigated in other cell types and in regard to disease specificity [34]. Vice versa, steroids can also control the circadian rhythm of cells, e.g., in the testicles, heart, fibroblasts, etc. [35,36,37]. Asthma and COPD studies on the circadian rhythm of GR activity are lacking, even though they were indicated by several investigations [38,39] (Figure 1).

Only for asthma did a few studies report the effect of the circadian rhythm on the availability and function of the GR. Over 25 years ago, evidence was presented that the binding capacity and signaling of the GR is reduced in patients with nocturnal asthma, but hardly any study followed up to gain more details of this condition [39,40]. The same investigators presented a similar link between the circadian rhythm of GR function and steroid insensitivity in macrophages [41]. In turn, asthma medications, including steroids and β2 agonists, can modify the circadian rhythm and thereby alter the drug’s efficacy in vitro and in vivo [42]. In mice, the optimization of drug application in line with the circadian rhythm showed improved response [43]. In human epithelial cells, the circadian rhythm was decisive for their response to steroids via the GR [44]. However, such precise timing of medication will require strict co-operation of the patients.

Referring to the different GR isoforms that can be derived from the NR3C1 gene, some alleles have been reported to affect the circadian response to steroids [10]. Moreover, both the innate and the adaptive immune response are controlled by a circadian rhythm through the action of the GR [45]. It remains unclear if these findings, which were mainly obtained in isolated cells or animal models, can be translated 1:1 to humans [46]. It should also be investigated if all GR isoforms interact in the same way with those regulatory and transporting proteins that have been described for the function of GRα. Do all GR isoforms undergo control via the circadian rhythm? Only a few of the NR3C1 gene alleles have been assessed for functional differences, and these studies are mainly based on theoretical changes of function based on protein modeling.

## 4. The Chromatin Structure Regulates Steroid Response

The chromatin, which regulates the access of transcription-regulating proteins, such as the GR, to the DNA, does not have a fixed structure and undergoes continuous conformational changes. These structural changes of the chromatin can be affected by the microenvironment of cells or organisms, including the composition of the extracellular matrix, the presence of inflammatory cells, cigarette smoking, exposure to chemicals, etc., as well as by other conditions, such as pregnancy, diseases, or medication [47,48,49,50].

**Figure 2:**
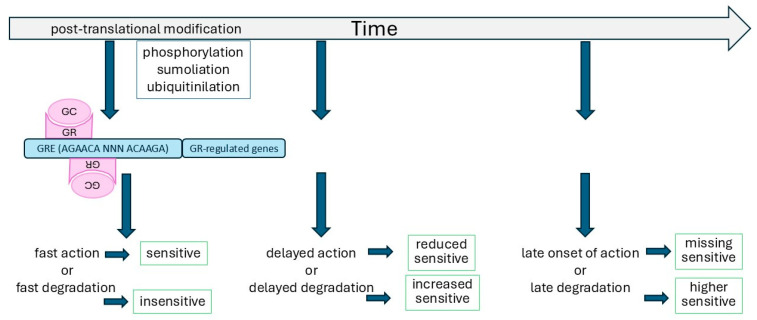
Timing of post-translational GR modification on steroid sensitivity.

Interestingly, exposure to some of the risk factors for chronic inflammatory diseases during the embryonic stage is indicated to pre-set the development of asthma or COPD during adult life [49,50,51,52,53,54]. Another important factor that can modify the function of steroids is the location of the GRE in the context of other transcription factor binding sites. It was implicated that the position of the GRE within the three-dimensional structure of the local chromatin affects the transcriptional efficacy of the GR [51]. The GR’s binding strength to a specific GRE is affected by the location of this GRE within the structure of the chromatin or by the distance to other transcription factor binding sites [52,53,54]. At least in vitro, the effects of the GR can be memorized over some time in primed cells by an unknown mechanism, and this might explain partly how reduced or enhanced steroid sensitivity is handed down over generations [55,56,57,58,59]. The reason why some conditions, such as modified chromatin structures, are inherited or not remains to be discovered [57,58,59,60]. Some data suggested that the timing of the priming event might be important, but the precise mechanism remains elusive, and it is unclear whether such events are reversible in the next generations (Figure 2).

The changes of the chromatin and, therefore, the GRE are regulated by the phosphorylation, sumoilation, ubiquitinilation, etc., of histones as well as transcription factors. The effects of these protein structural and functional changes depend on the timing of each event [61,62]. The interaction between the GR and the chromatin structure of its target genes had been summarized by Lovem et al., and it was concluded that there are many gaps of knowledge to be filled before we will be able to understand these mechanisms, which might be target gene specific [63]. Furthermore, the binding of the GR to the GRE might alter the structure of the chromatin in a broader area and affect its own binding capacity and the transcription of neighboring genes, probably in a cell-type or tissue-specific manner [64,65,66]. This ongoing change in the chromatin structure alters the position of transcription factor binding sites to each other, including that of the GRE and, thus might modify its interaction or accessibility to the GR, leading to altered steroid sensitivity [64,65,66].

Some data suggest that the cell-type specific regulation of steroid-regulated genes is defined by the structure of the chromatin, as was indicated in other diseases [62,67,68]. None of these changes of the local chromatin structure that regulate the access of the GR to the GRE or other transcription factor binding sites have been analyzed regarding the kinetic of steroid response in a disease-specific manner. Nevertheless, the available data indicates that small changes in these regulatory mechanisms might cause tissue structural changes, and they can also alter the sensitivity to steroids and, therefore, they need to be investigated.

Another problem in conducting such investigations might be the uncertain definition of chronic inflammatory lung disease stages [69,70,71,72,73]. Small changes in the kinetics of such protein modifications can affect the function of cells and the response to hormones and medication. Theoretically, such changes in the kinetic of any protein that is involved in the GR structure/function regulation can lead to reduced or increased steroid response, and corresponding studies should be conducted.

## 5. GR Isoforms: Their Origin and Regulation

The impaired function of the GR has been explained by modified DNA sequences of the encoding NR3C1gene, and several NR3C1 alleles have been linked to reduced steroid response; however, many steroid-insensitive patients do not carry such a mutation [74,75]. Some of the NR3C1 alleles cause alternative transcription start sites, which resulted in five different mRNAs and corresponding GR proteins [20,75,76,77] (GRα, GRβ GRγ GR-A, GR-P). In asthma alone, a further 21 GR isoforms have been described [76], but it remains unclear when and why the different GR isoforms occur. There is even less information about the functional consequences of these GR isoforms. One report suggested that different GR isoforms occur in a tissue or cell-type specific pattern and exert distinct transcriptional activity [77].

There are more variations of the GR protein resulting from alternative translation; at least for GRα, seven such variants have been described (GRα-B, -C+, C2, C3, D1, -D2, -D3) [20,78]. Importantly, the regulation of the expression of these different GR protein isoforms is not well understood but is certainly not based on known genetic alterations. The action of the GR on inflammation and remodeling depends on its interaction with the GRE and other transcription factors, as well as with their binding sites on different target genes [78,79]. Taking into account that we do not understand the role of chromatin formation in time and location; it is possible that genetic alterations of certain DNA sequences might play a regulatory role and thus impact steroid sensitivity [80,81,82].

Some studies implied that the expression of these GR isoforms is regulated by condition, cell type, or circadian rhythm [83,84]. It remains to be studied if the regulation and function of these GR isoforms are linked to specific conditions or kinetics. Most studies suggested that the transcriptional function of GRα is hindered by competitive GRE binding through other GR isoforms [85,86,87].

Specific splicing of GR mRNA to form GRβ can be directed by other hormones, such as dehydroepiandrosterone [86]. There is evidence that the transcription of GR splicing variants changes during development and thus, steroid sensitivity might change from juvenile to adult life [88]. GRγ shares 94% homology with GRα and has an additional amino acid within the DNA binding site [86]. GR-A lacks exons 5–7 and, therefore, its ligand binding is reduced; it has been described to be expressed higher in some areas of the cortex when compared to GRα [87]. This finding implies that studies should be conducted on age-dependent GR expression by application of transcriptomic analyses. In an animal model, the splicing isoform, GR-P, has been reported to modify the function of GRα in a cell-type specific manner using [89,90]. In humans, the expression of GR-P occurred mainly in the placenta and was altered by maternal and embryonic conditions [89,90,91].

Furthermore, 21 GR isoforms have been described in the lymphocytes of healthy probands and asthma patients, but their link to steroid responsiveness is unknown [78]. Other data implied a sub-cellular specific distribution and function of GR isoforms and, therefore, an organelle-specific steroid response. GRγ has been associated with steroid effects on mitochondria activation and modified cellular energy levels [92]. Children with acute lymphoblastic leukemia showed reduced clinical steroid response, and in isolated cells, steroids up-regulated GR-γ expression over 10 h, while it returned to baseline expression afterwards in probands with normal clinical response [93]. In the same patient group, it was shown that the GRγ-isoform had approximately 50% GRα activity and was assumed to be the cause of reduced steroid response [94]. Assuming that the transcription and translation of any GR isoform is delayed or advanced compared to GRα, this will affect the availability and function of the GR signaling cascade in a disease or cell-type specific manner, but such studies are missing [95].

**Figure 3:**
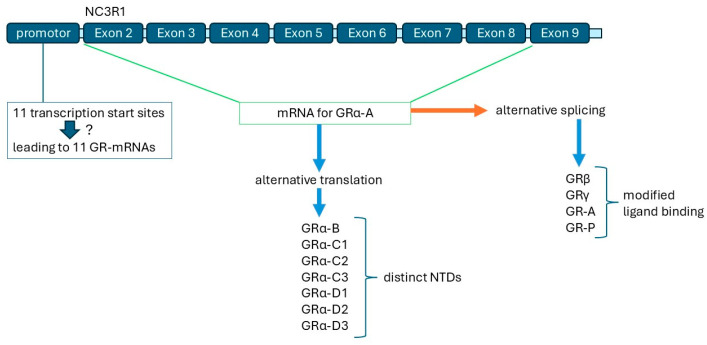
NC3R1 gene structure and its implication on GR isoforms (adapted from Ref. [20]). GR isoform expression was suggested to depend on time and affect its function [93,94]. Distinct translation isoforms vary in the length of the N-terminal domain (NTD). Only one insertion was reported for the DNA-binding domain of the GR. Blue arrows indicate resulting GR-isoforms, the question mak indicates an unknown mechanism, the orange arrow indicates a distinct mechanism of RNA processing.

Furthermore, GRα alone can be modified at the post-translational level into seven different structures, mainly affecting the N-terminal domain. Moreover, the structure and function of the GR can be altered post-translationally by phosphorylation, sumoilation, and/or ubiquitinilation [95,96,97]. The modification of different phosphorylation sites within the GR protein led to distinct activities regarding the target promoter, but the kinetic of these modifications has not been well studied [89]. Regarding the distinct GR isoforms, mainly the role and modification of the GRβ isoform but not that of the other isoforms have been investigated regarding steroid insensitivity in the context of cell-type specific and kinetic activity [97,98]. Referring to the above-named GR isoforms resulting from either different transcription start sites, mRNA splicing or post-transcriptional modifications would be of interest in assessing whether their expression follows a circadian rhythm (Figure 3).

## 6. GR Interaction and Regulation by Other Transcription Factors

Alternatively, or in addition to the above-described direct action as a transcription factor, the GR forms a complex with another transcription factor, which modifies the binding of these to their specific GRE DNA sequences [20]. For example, a single ligated GR inhibits the transcription-inducing effect of pro-inflammatory transcription factors by forming a complex with them, e.g., NFkB (nuclear factor ‘kappa-light-chain-enhancer’ of activated B-cells), AP-1 (activator protein-1), C/EBPs (CCAAT enhancer-binding proteins), or CREBP (cyclic AMP-binding protein) [99,100,101,102,103]. The GR can bind other free transcription factors or those that are already bound to their specific DNA sequence and thereby lower inflammation [21,22,23]. This complex formation can occur in either the cytoplasm or in the nucleus [21,22,23]. However, the precise mechanism by which the GR limits the action of other transcription factors, especially of those already bound to their promoter sequence, is unknown for most and could result from steric hindrance blocking the access of the transcription machinery or affecting the three-dimensional structure of the surrounding chromatin [104,105,106]. It can be speculated that such events are disease specific and alter steroid response.

The GR also controls cell proliferation, which contributes to the limitation of cell growth and is important to tissue remodeling in asthma. In isolated airway smooth muscle cells of healthy probands, the synchronization of GRα and C/EBP-α activity was achieved by the combination of steroids with β2 agonists, resulting in enhanced production of the anti-proliferative protein p21 (Waf1/Cip1) [107]. In human lung macrophages and epithelial cells, a similar effect of β2 agonists was observed after inhalation of the drug alone or with steroids, which reduced the activation of pro-inflammatory signaling pathways [108,109,110,111,112]. However, in airway smooth muscle cells of asthma patients, the anti-proliferative effect of combined drugs was not observed, while the anti-inflammatory effect occurred at a slower path compared to healthy controls [113]. Furthermore, the timing of the GRα—C/EBP-α interaction might be affected by infection, cell environment, or inflammatory conditions [108,109,110,111,112,113].

Moreover, the GRα can be activated by β2 agonists or the mineralocorticoid receptor, independent of ligation, and this alters the timing of its activity [114,115]. Other factors, such as cell density, circadian rhythm, and nutrition status, might affect the action of the GR, as had been implied by in vitro experiments in human primary cells [111,116]. Most GR ligands induced a fast translocation into the nucleus within 15–30 min [117], but some have been reported to need more time due to an unknown hindering mechanism [111,112,113,118]. Thus, it remains to be investigated if the kinetic of these interaction diseases specifically regulates the active phase of the GR and its complex formation with co-transcription factors, as was suggested by in vitro data [118,119,120].

Reduced steroid sensitivity correlated with the severity of asthma [121] and with a history of smoking [122]. Regarding tissue remodeling, mechanical stress, and the resulting reduced efficacy of steroids in COPD patients, the timely interaction with other proteins might be important and remains to be studied at different disease stages [123,124]. By which mechanism COPD patients can acquire reduced steroid sensitivity in lung immune cells and circulation lymphocytes remains unclear and might even vary between patients [7,11]. Reduced immune response by the GR can be acquired during the cause of COPD through either interacting the GR with Th2 high-inflammatory signature (TAC1) proteins or by regulating its expression; thus, it is difficult to say which event is first [24]. Furthermore, the effect of steroids on TAC1 regulation by the GR depends on the promotor structure of the TAC1 gene [125].

## 7. Steroid Availability and Its Regulation

Despite the above-described structural alterations of the GR protein, other mechanisms can affect the timely availability of steroids. It is assumed that only freely circulating steroids can penetrate the cell wall and exert their intracellular action [126,127]. Circulating steroids are bound corticosteroid-binding globulin (CBG), but the regulatory mechanism underlying the balance of CBG-bound and free steroids is not well understood and has not been reinvestigated in recent years [128,129]. Genetic variants of CBG have been described, but their effect on the ratio of free and bound steroids is not well determined [130] and is affected by the presence of pro-inflammatory cytokines, such as interleukine-6 [131]. However, the details and timing of these regulatory processes remain unknown.

In the context of steroid insensitivity, it has to be noted that inflammatory conditions reduce CBG fast and result in increased levels of free steroids [132]. It was reported that different synthetic steroids bind with different strengths to CBG and are also released in a drug-specific pattern [130], but whether these differences are linked to steroid insensitivity remains to be studied. Furthermore, CBG is also known as either transcortin and is encoded by the gene SERPINA6, by which several variants with varying steroid-binding strength have been described [133,134,135,136,137]. A recent study suggested a link between SERPINA6 protease imbalance and COPD in the peripheral lung [138].

Although the binding and release of steroids to CBG is of importance to understanding steroid response during therapies, studies assessing the timing of steroid release or binding to CBG in distinct diseases are missing. There is also evidence that CBG deficiency can be transmitted from mother to child during pregnancy [139]. This finding might imply a novel epigenetic trait for steroid sensitivity but is at this stage only hypothetical. A recent review of CBG, its regulation, and its role in steroid function has been published by Hammond [140].

## 8. Discussion

Large-scale studies to define the precise timely function of all GR variants and modifications are needed if we want to develop personalized therapies for asthma, COPD, and other chronic inflammatory diseases, not only of the lung. Furthermore, such studies will help us to better understand the role of the GR in physiology and pathophysiology. Is it thinkable that the interaction of the GR with its complex-forming partner proteins underlies an unknown circadian regulation pattern and that this is off balance in chronic inflammatory lung diseases? Small changes in the timing of one of the components of the GR expression or of its signaling cascade might then have consequences for steroid effects and thus reduced steroid response.

To understand the complex nature of the GR network as the cause of reduced steroid sensitivity in chronic inflammatory lung diseases, studies of the precise timing of the GR’s location, activity status, and complexing partner proteins are implicated. Such studies must include the aspect of the many GR SNPs and GR isoforms.

Because each GR isoform and NR3C1 gene SNP might interact differently with other regulatory proteins over time and in specific conditions, the task will require the inclusion of genomic, transcriptomic, proteomic, and post-transcriptomics, as well as protein complex studies. Such studies will require large networks, manpower, and money to understand the networking of the GR. The promotor of the NR3C1 gene has eleven potential transcription start sites, and the GRα-A mRNA can be spliced and translated into seven GR protein variants, which is equal to seventy-seven structural different GR proteins [20]. These are subject to an uncertain number of post-translational modifications that might affect the activity status, location, or function of the GR [22,23,24,25,26,27,28,141,142,143]. It is also uncertain if only the GRα is subject to splicing, or if all other GR isoforms can also generate further variants with different functions, including steroid sensitivity. Thus, the number of study subjects that have to be included in ‘all-GR analysis’ would be enormous. These facts show the necessity of pre-selecting patients with well-defined specific GR variants and then analyzing the kinetics of expression, activity, and tissue or cell-type specific expression.

**Figure 4:**
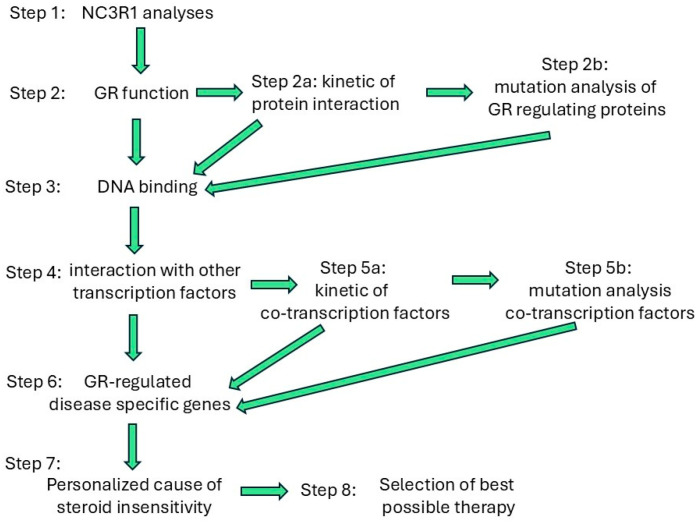
Concept to determine the cause of individual steroid insensitivity and therapy.

However, such an undertaking will gain new knowledge that provides not only a better understanding of the pathogenesis of chronic inflammatory lung diseases but it will also provide a better understanding of our physiology and of the cause of other diseases. Thereby, it will open new avenues for individual medicine and better therapies. The timing of therapeutics has been reported to improve the beneficial effects of steroids and other asthma drugs via GR function; unfortunately, these promising concepts have not yet been adapted as therapeutic strategies. To successfully apply such therapeutic regimes, it will also be necessary to explain the benefits to each patient (Figure 4). Furthermore, new methods for monitoring such therapies will be needed, a problem that has been assessed by others [144].

## 9. Conclusions

In summary, the effect of the nuclear GR largely depends on the presence of additional proteins including those that control its phosphorylation, degradation, structure, cytosol–nucleus transportation, and interacting transcription factors. All these interacting factors need to be present at a specific time and intracellular location to facilitate the GR action. A delay in any of them might lead to a lower GR action or failure and lead to chronic diseases. Unfortunately, such investigations are rare or missing.

## Data Availability

No original data used.

## Abbreviations

The following abbreviations are used in this manuscript:

AP-1	Activator protein 1
CBG	Corticosteroid-binding globulin
C/EBP	CCAAT enhancer-binding protein
COPD	Chronic obstructive pulmonary disease
CREBP	Cyclic AMP-binding protein
DNA	Deoxyribonucleic acid
DOAJ	Directory of open-access journals
FKBP	FK506-binding proteins
GC	Glucocorticoid
GR	Glucocorticoid receptor
GRE	Glucocorticoid response element
HSP	Heat shock protein
mRNA	Messenger ribonucleic acid
NFkB	Nuclear factor ‘kappa-light-chain-enhancer’ of activated B-cells
SNP	Single-nucleotide polymorphism
TAC1	Th2 high-inflammatory signature protein 1
